# Effectiveness of nursing educational interventions in managing post-surgical pain. Systematic review

**DOI:** 10.17533/udea.iee.v37n2e10

**Published:** 2019-09-19

**Authors:** Antonio Reaza-Alarcón, Beatriz Rodríguez-Martín

**Affiliations:** 1 Nurse. Universidad de Castilla-La Mancha, Facultad de Ciencias de la Salud, Talavera de la Reina (Toledo), Spain. Email: areazal96@gmail.com Universidad de Castilla-La Mancha Spain areazal96@gmail.com; 2 Nurse, Masters, Ph.D. Associate Professor, Universidad de Castilla-La Mancha, Facultad de Ciencias de la Salud, Talavera de la Reina (Toledo), España. Visiting Researcher; University College Dublin, Ireland. Email: beatriz.rmartin@uclm.es Universidad de Castilla-La Mancha Facultad de Ciencias de la Salud España beatriz.rmartin@uclm.es

**Keywords:** effectiveness, nursing research, pain, postoperative, pain management, patient education as topic, review., efetividade, pesquisa em enfermagem, dor pós-operatória, manejo da dor, educação de pacientes como assunto, revisão.

## Abstract

**Objective.:**

Analyze and integrate studies that inquire on the benefits of nursing educational interventions to manage post-surgical pain.

**Methods.:**

A systematic search was conducted in the databases of Scopus, Medline (Pubmed), Web of Science, The Cochrane Library, and CINAHL of systematic reviews, randomized clinical trials, and quasi-experimental studies published in English and Spanish until 2018 that analyzed the effectiveness of educational interventions in managing post-surgical pain in adult patients.

**Results.:**

Twelve studies complied inclusion criteria, of which nine reported less pain in the group receiving the educational intervention. These interventions also helped to diminish the level of anxiety and improved functionality to perform activities of daily life. The level of quality of the studies was medium.

**Conclusion.:**

Although the review showed that nursing educational interventions could influence on the relief of post-surgical pain, more rigorous studies are necessary, with bigger sample sizes and higher methodological quality, which help to establish the real effectiveness in managing post-surgical patients with pain.

## Resumo

**Objetivo.:**

Analisar e integrar os estudos que indagarão nos benefícios das intervenções educativas de enfermagem para o manejo da dor pós-cirúrgico.

**Métodos.:**

Se realizou uma busca sistemática nas bases de dados Scopus, Medline (Pubmed), Web of Science, The Cochrane Library e CINAHL de revisões sistemáticas, ensaios clínicos aleatorizados e estudos quase-experimentais publicados em inglês e castelhano até 2018 que analisassem a efetividade das intervenções educativas no manejo da dor pós-cirúrgico em pacientes adultos.

**Resultados.:**

Doze estudos cumpriram critérios de inclusão, dos quais nove reportaram menos dor no grupo que recebeu a intervenção educativa. Estas intervenções também ajudaram a diminuir o nível de ansiedade e melhoraram a funcionalidade para realizar as atividades da vida diária. O nível de qualidade dos estudos foi médio.

**Conclusão.:**

Embora a revisão mostrou que as intervenções educativas de enfermagem poderiam influir no alivio da dor pós-cirúrgico, são necessários estudos mais rigorosos, com maiores tamanhos de amostras e de maior qualidade metodológica, que ajudem a estabelecer a real efetividade no manejo do paciente pós-cirúrgico com dor.

## Introduction

Pain is a serious public health problem of global relevance. The International Association for the Study of Pain defines pain as an unpleasant emotional and sensory experience associated to current or potential tissue damage. By being a subjective experience, its measurement varies according to the person’s perception.(1) Besides, according to its nature, pain can be acute, produced by tissue damage, of sudden onset and which ceases with the passage of time; or chronic, when it occurs during a long period of time, causing a limiting problem in daily life and which is aggravated by factors, like age, gender, and environmental and psychological factors, among others.(1) In the recent decades, knowledge has increased on the physiopathology of pain. However, evidence shows that its treatment continues being poor and insufficient. In this sense, postoperative pain is still a challenge in the management of post-surgical patients, having important physiological, psychological, economic and social consequences.(2) Prior studies show that over 80% of patients subjected to a surgical process experience pain. Of this percentage, 75% of patients experience moderate, severe, or extreme pain.(3,4) Other studies indicate that nearly half the patients have severe pain due to inadequate healing.(5,6) 

It is known that in the perception of pain, multiple factors influence, such as the type of intervention, age, gender, or the patient’s own expectations, which hinders foreseeing the level of pain patients can experience after surgery.(7) Prior research show that efficient post-surgical control contributes to facilitating the patient’s physical and psychological recovery, diminishes the hospital stay, and improves quality of life and levels of stress. Also, reducing social and health costs.(8) Furthermore, nursing professionals are trained to educate patients in the control and management of pain. We know that one of the obstacles to manage effectively post-surgical pain is the patient’s lack of knowledge or misunderstandings. Thereby, educational interventions can impact upon patients by modifying their behavioral pattern, knowledge, attitudes, and skills to achieve improved health. Hence, good preoperative information, provided by nursing professionals, can help to prepare patients for the postoperative phase, equipping them with autonomy to become active components of their care and treatment, which will contribute to better managing pain.(1) Most prior studies have focused on relief of post-surgical pain and management of anxiety. 

To our knowledge, no prior systematic reviews exist that additionally analyze other variables, like the development of daily activities or the vital constants. The objective of this review is to analyze and integrate studies that inquire on the effects of nursing educational interventions on the relief of post-surgical pain. 

## Methods

This study conducted a systematic review of randomized clinical trials (RCT), quasi-experimental studies, systematic reviews and meta-analyses that analyzed the effectiveness of educational interventions, conducted during the preoperative and postoperative phases, on the relief and prevention of post-surgical pain. The search included articles published in English and Spanish in the databases of Scopus, Medline (Pubmed), Web of Science, The Cochrane Library, and CINAHL. It included articles to February 2018 to analyze studies reflecting the association between the educational intervention and relief and decrease of post-surgical pain in patients subjected to any type of surgery. Table 1 gathers the search strategy used. In addition, a secondary search was conducted through the references cited by the studies found in our initial search and which were related with the principal objective of the study. 

**Table 1:** Search strategy used in the databases analyzed

Database	Search strategy
Web of Science	
MedLine (PubMed)	(pain OR acute pain) AND (“post surgical” OR postoperative OR surgery) AND (“pain
The Cochrane Library	management” OR “Pain control” OR “Pain relief” OR “pain education” OR “patient
CINAHL	education”) AND (educati* OR intervention) AND (nurs*)
Scopus	

Two reviewers performed independently the search and selection of the articles; thereafter, agreeing on the results. This process used the following criteria. Inclusion criteria: 1) Systematic reviews, RCT, quasi-experimental studies, and meta-analyses inquiring on the effectiveness of educational interventions in managing post-surgical pain; 2) Studies published to February 2018 in English or Spanish; and 3) Studies including in their sample a population over 18 years of age. Exclusion criteria: 1) Studies showing very low quality after the evaluation with instruments for their analysis (score < 40% of the instrument’s maximum score); 2) Educational interventions aimed at the relatives; and 3) Studies including in their sample patients with mental pathology. The study followed the principles of the PRISMA Declaration.(9) 

To analyze the quality of articles potentially eligible, the following instruments were used: Checklist for Systematic Reviews and Research Syntheses from the Joanna Briggs Institute and AMSTAR to evaluate the systematic reviews, the JADAD scale to evaluate the RCT, Joanna Briggs Institute Checklist for Quasi-experimental Studies to evaluate quasi-experimental studies and the Newcastle-Ottawa Scale (NOS) to evaluate the quality of the meta-analyses. The AMSTAR scale has 16 items with four possible responses.(10) The Checklist for Systematic Reviews and Research Syntheses is an instrument to evaluate the quality of systematic reviews and has 11 items with 11 being the maximum score.(11) The NOS has eight items, with a star granted in each item referring to the categories of selection and exposition, and a maximum of two in the comparison.(11) The JADAD scale has five items, with a score from 0 to 5, considering those aspects related with the study bias, like randomization or masking.(12) The quasi-experimental studies were evaluated with the Joanna Briggs Institute Checklist for Quasi-experimental Studies, which has nine items, with 9 being the maximum score.(13) 

## Results

**Figure 1:**
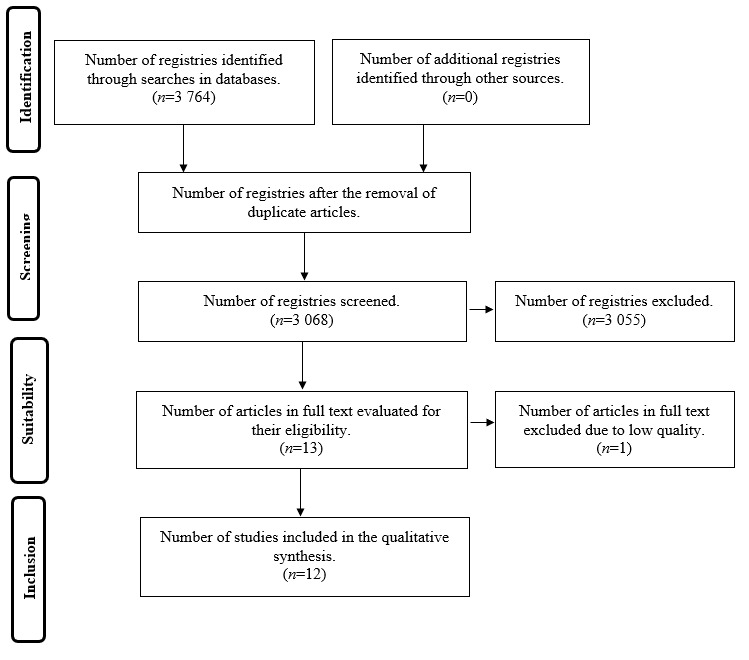
Flow diagram of the search and selection process

The initial search in the databases selected found 3 068 articles after eliminating articles duplicated in various databases. After a first review of the titles and abstracts 3055 articles were eliminated because of dealing with themes different from the object of study. Thereafter, 13 articles were reviewed in full text, from which one article was excluded because it had an insufficient score after its quality analysis (Figure 1). Finally, 12 articles were included in this review. Table 2 summarizes the principal characteristics of the studies analyzed.

In relation with the type of study, six of the articles analyzed were controlled randomized trials (CRT),(14-19) a systematic review and meta-analysis(20) and five quasi-experimental studies.(21-25) Regarding the geographical area, the studies were conducted in seven countries: South Korea,(14) China,(15,17,21,25) Canada,(16) Finland,(18) Norway,(19) Spain,(22) the United States (23), and Holland.(24) The studies included analyzed the effectiveness of different educational interventions aimed at the management and relief of post-surgical pain. In this sense, some of the studies analyzed the effectiveness of educational interventions online,(1,3) others on educational interventions aimed at managing pain and anxiety;(2,4,5,7) certain studies inquired on the relationship between knowledge on the consumption of opioids and the pain management,(6,8,10,11) while others analyzed the effectiveness of an educational intervention in ambulatory surgery (22) or the relationship between pain and post-surgical rehabilitation.(12)

Furthermore, certain heterogeneity was found regarding the types of interventions used in the studies, finding the following interventions: educational interventions based on the delivery of graphic material,(15,17,19,22,25) use of audiovisual material and new technologies (14,16,24,25) as support to the educational intervention and interventions based on informative talks.(18,21,23) In all cases, the educational intervention was directed by the patients, except in one of the studies(14) in which the intervention was aimed at both patients and nursing professionals. 

The principal result measurements analyzed in the studies were pain, anxiety, or level of knowledge about the patient’s pain. As secondary results, consumption of analgesics, functional rehabilitation, quality of life, vital constants, or functional disability associated with pain were analyzed.(15,16,22,24,25) In relation with the scales and instruments used by the studies to measure the results, the studies included used the Numerical Graduation Scale,(14,16,19,23-25) the Visual Analog Scale (VAS),(15,18,21,22) the Brief Pain Inventory-Short Form (BPI-SF) scale (17)^1^ and the State-Trait Anxiety Inventory (STAI).(15,18,20,21)

**Table 2:** Principal characteristics of the articles analyzed

Reference	Summary of the study
14	Authors; year: Hong and Lee; 2014. Country: South Korea. Type of study: Randomized Clinical Trial (RCT). Objective: To analyze the relationship between an intervention that uses an evidence-based online educational guide and improved levels of pain in patients intervened from an abdominal surgery. To know the nursing staff’s level of knowledge about pain. Intervention: A control group (CG) was established of patients, which did not receive information about the surgery, and two experimental groups (EG1 and EG2) that did receive said information. After the surgery, tests were conducted to evaluate the level of pain and of knowledge about it by the patients. For the nursing professionals, a pre-post test was conducted after the educational intervention to assess the level of knowledge. Characteristics of the sample and follow up: 112 patients over 19 years of age, intervened for a surgery lasting over one hour, with degree 1 or 2 in the body classification of the American Association of Anesthesiology, conscious patients, capable of communicating and oriented, with stable vital signs before the operation. Pain was measured at one hour, 6, 12, 18 and 24 hours. From the first day after surgery to the 14th day, pain was measured in the EG1, and from the 15th day to the 18th day, it was measured in the EG2. The sample of professionals was comprised by 27 nursing professionals who had worked at least one year in a pre-operative anesthesia unit. Knowledge of the nurses was measured on the day of the intervention, at 14 days and 28 days later. Result measurements: Numerical Graduation Scale of pain. Results: After the educational intervention aimed at the nursing staff, the score between the pre- and posttest increased. Patients in the EG showed lower scores of post-operative pain with respect to the CG. Conclusions: Educational interventions using evidence-based online guides improve the level of post-operative pain in patients and increase the level of knowledge of the nursing staff. In spite of the aforementioned, the presence of contradictory evidence and the lack of access to scientific evidence are factors that hinder the elaboration of these guides. Implementation of these guides in the clinical practice can be useful to reduce postoperative pain; further research is necessary investigations to contribute with more information to this theme. Quality score: 2/5 in the JADAD scale.
15	Authors; year: Lee et al.; 2017. Country: China. Type of study: RCT. Objective: To determine the effects of an educational intervention about pain and anxiety in patients intervened for spinal surgery. Intervention: The control group (CG), which included 43 patients, received a verbal 10-minute educational intervention on the day before the surgery. The experimental group (EG), which had 43 patients, received a pamphlet with information about the characteristics of pain, the surgical procedures and its care, additionally, it received a 40-minute educational intervention. Characteristics of the sample and follow up: 66 patients over 20 years of age who knew Chinese Mandarin or Taiwanese and without hearing or visual difficulties. The variables to study were measured one day before the surgery, half an hour before the surgery, and the day after such. Result measurements: Visual Analog Scale (VAS) to evaluate pain. State-Trait Anxiety Inventory (STAI) scale to evaluate anxiety. Results: After an intervention based on the combination of delivering a pamphlet with information about the characteristics of pain, the surgical procedures and its care, and a 40-minute educational intervention, the patients had lower levels of pain and anxiety than the patients in the control group. No significant changes were found in the vital constants before and after the intervention. Conclusions: Educational interventions help significantly in lowering anxiety and post-operative pain. However, these seem to have no repercussion on variables, like blood pressure, cardiac frequency, and respiratory frequency. The use of these types of interventions in the clinical practice can be useful to manage patient anxiety. Quality score: 3/5 in the JADAD scale.
16	Authors; year: Martorella et al.; 2012. Country: Canada. Type of study: RCT. Objective: To analyze the effectiveness of an educational intervention via online on the relief of post-surgical pain in patients intervened for heart surgery. Intervention: The CG had 30 participants who received habitual education before the surgery. The EG had 30 participants who received the habitual intervention before the surgery plus online intervention, where the definition of pain was detailed, how could affect each patient, and advice on its treatment. Characteristics of the sample and follow up: 60 patients over 18 years of age intervened for the first time for heart surgery, capable of understanding and communicating in French. The educational intervention of both groups was conducted days prior to the surgery, and the subsequent results were measured through questionnaires on the day of hospital admission, one day after the surgery and on the seventh day after the surgery. Result measurements: Numerical Graduation Scale of pain. Results: After the educational intervention online, no significant differences were found between the groups in the level of pain perceived after the surgery. However, the experimental group receiving additional information online had less interferences in their daily life. Besides, significant differences were found in the difficulties of patients during deep breaths, when coughing or in appetite. Also, participants from the EG showed a change in behavior about taking analgesics. During the measurements, the experimental group showed less pain, but it was not statistically significant. Conclusions: An educational intervention online can influence positively on the pain management and can provoke a positive change in behavior upon such, diminishing barriers in its management and the influence of pain in daily life activities. These types of interventions can be useful in the clinical practice to conduct health education in the post-operative setting. Further research is needed for in-depth exploration of its effects. Quality score: 2/5 in the JADAD scale.
17	Authors; year: Guo et al.; 2012. Country: China. Type of study: RCT. Objective: To determine the effects of an educational intervention on reducing anxiety, depression, or pain and improving patient recovery. Intervention: The CG had 77 patients who received the habitual educational intervention, which consisted in verbally informing one day before the surgery of the surgical process, risks, analgesia, and pain management. The EG had 76 patients who received a broader session two or three days before the intervention, complemented with a specific pamphlet explain the pre-operative preparation, the stay in the Intensive Care Unit, and the recovery at home. Characteristics of the sample and follow up: 153 patients over 18 years of age who would be subjected to heart surgery, able to communicate in and understand Chinese Mandarin. After the surgical intervention, a follow up was conducted of the patients during the first seven days after the surgery. Result measurements: To measure the pain perceived and its influence on daily life, the Brief Pain InventoryShort Form (BPI-SF) scale was used. Results: After an educational intervention complementing the habitual information with additional specific information, a significant decrease of anxiety was reported in the experimental group with respect to the control group. In relation to pain, no significant differences were found among patients from both groups. However, the control group had lower levels of pain and less problems to fall asleep. Conclusions: Specific educational interventions for cardiac patients who will be subjected to surgery help patients psychologically, diminishing their anxiety and depression. However, these interventions do not affect significantly the relief and management of post-surgical pain. These types of interventions are easy to design and carry out. In the clinical practice, these interventions can help to reduce the pre- and post-operative anxiety of patients. Quality score: 3/5 in the JADAD scale.
18	Authors; year: Kesänen et al.; 2017. Country: Finland. Type of study: RCT. Objective: To analyze the effectiveness of an educational intervention on anxiety, quality of life, disability, and pain in patients intervened for spinal stenosis. Intervention: The CG had 50 patients who received the habitual education consisting of information on the type of intervention, complications, different treatments, etc. The EG had 50 patients who received more detailed education via telephone where patients were also encouraged to become an active part in their treatment, aside from the habitual intervention. Characteristics of the sample and follow up: 100 patients 18 years old or over, with capacity to communicate and understand the local language (Finnish), with capacity to use a mobile phone and who would be subjected to spinal surgery. Before the surgery, knowledge of both groups was evaluated. The variables to study were registered upon admission, after the operation, and after three and six months of the operation. Result measurements: VAS to evaluate pain and Spielberger’s State Trait Anxiety Inventory (STAI Form Y-1) to evaluate anxiety. Results: After an educational intervention, based on empowering patients, its inclusion in the treatment and on the dissemination of the habitual information via telephone, a significant increase was found of patient knowledge on the characteristics of postoperative pain. Although a significant drop of anxiety was reported in the experimental group after the surgery, no significant differences were found in both groups. There was a decrease in pain relief in both groups, without significant differences between both. Pain relief was greater in the first three months after the intervention. Conclusions: An educational intervention based on empowering patients and on the dissemination of the habitual information via telephone, by itself does not significantly affect relief of post-surgical pain, quality of life, or disability in patients subjected to spinal surgery. In spite of the aforementioned, this type of intervention is effective to diminish anxiety. Quality score: 4/5 in the JADAD scale.
19	Authors; year: Bjørnnes et al.; 2016. Country: Norway. Type of study: RCT. Objective: To determine the characteristics of pain, consumption of analgesics, and the impact of an educational intervention on managing post-surgical pain in patients subjected to heart surgery. Intervention: The CG had 175 patients, who received a 10-minute talk that solved possible doubts about the surgical process and pain relief. The EG had 174 patients who received in complementary manner a brochure that indicated the importance of relieving post-surgical pain, how and when to seek help after the surgery, pharmacological and non-pharmacological methods, and common problems after the surgery. Characteristics of the sample and follow up: 349 patients over 18 years of age, able to read and write in the local language and who were programmed for specific cardiac surgery. After delivering the pertinent information to each group, the variables to study were measured 24 hours before the surgery, and follow up of the sample was conducted on day 10 after the surgery, as well as 1, 3, 6, and 12 months after such. Result measurements: Numerical Graduation Scale of pain. Brief Pain Inventory-Short Form (BPI-SF). Results: After an educational intervention complementing the habitual talks with a brochure, no significant differences were found between the control and experimental groups regarding moderate-severe pain (p=0.6) during the follow up, or in the interferences in daily life (p=0.3) in the first two weeks. Conclusions: The results of this study do not confirm the effectiveness of an educational intervention based on an informative brochure to relief post-surgical pain. Pain control continues being a problem in patients subjected to surgery, which why it is necessary for future studies to develop and assess educational strategies that aid in the relief of post-surgical pain. Quality score: 3/5 in the JADAD scale.
20	Authors; year: Ramesh et al.; 2017. Country: Canada, China, Iran, Greece, the United Kingdom, Norway, Thailand, and Australia. Type of study: Systematic review and meta-analysis. Objective: To analyze the benefits obtained after an educational intervention in patients subjected to heart surgery. Intervention: Multiple educational interventions aimed at patients subjected to heart surgery. Characteristics of the sample and follow up: 2 071 patients over 18 years of age subjected to any type of heart surgery. Result measurements: Levels of anxiety measured with the Zung Self-Rating Anxiety Scale and the StateTrait Anxiety Inventory. Level of pain evaluated with the Brief Pain Inventory-Short Form (BPI-SF) and with the Visual Analog Scale. Days of hospital stay were measured. Results: Of the variables studied, only in anxiety was a significant difference found between the control group and the experimental group that received the educational intervention. The other variables (pain, depression and hospital stay) revealed no statistically conclusive change. Evidence of the studies included was low due to the lack of clarity in the partiality and imprecision of the effects studied. Results obtained were not backed by studies of quality. Conclusions: Educational interventions improve significantly levels of anxiety of patients; however, these cannot exert significant changes in variables, like pain, depression, or days of hospital stay. In the clinical practice, educational interventions can be useful to treat anxiety and promote patient relaxation. More studies are needed and with higher methodological quality that test the effects of educational interventions in managing post-surgical pain. Quality score: JBI scale for Systematic Reviews 8/11.
21	Authors; year: Wong Eliza Mi-Ling et al.; 2010. Country: China. Type of study: Quasi-experimental study. Objective: To analyze the effects of an educational intervention about pain, anxiety, and independence in patients intervened for musculoskeletal trauma. Intervention: The CG had 62 patients who received habitual care. The EG had 63 patients who received habitual care plus an educational intervention, which consisted of a 30-minute talk with information about pain and relaxation and breathing strategies. Characteristics of the sample and follow up: 125 adult patients who knew Cantonese, who were ambulatory before the surgery and diagnosed with musculoskeletal trauma. Result measurements: Pain was evaluated with the VAS scale. Anxiety was evaluated with the STAI and independence with the Chinese version of the Self-efficacy Scale. Results: After the educational intervention, no significant differences were found in pain over time that could determine that the educational intervention caused a difference in managing post-surgical pain. However, during the hospitalization period, a statistically significant difference was found on the level of pain between the CG and the EG, especially on days 2, 4, and 7, which suggested a possible effect of the educational intervention. The results were possibly influenced by other factors, like social support, economic status, family environment, etc. Conclusions: The results of this study suggest that an educational intervention during hospitalization can be effective for pain management, although it is possible that the results may be due to the use of analgesics. After an educational intervention, a trend is shown for pain relief in patients intervened for musculoskeletal trauma, although not significant. It is feasible that nurses implement educational interventions for relief of post-surgical pain, and it is likely that these interventions are well-accepted by the patients. Quality score: JBI scale for Quasi-experimental Studies 7/9.
22	Authors; year: Font Calafell et al.; 2011. Country: Spain. Type of study: Quasi-experimental study. Objective: To evaluate the efficacy of an educational intervention, conducted by nursing, based on the delivery of graphic material, to relieve post-surgical pain in patients intervened for hernia in a major surgery ambulatory unit. Intervention: The CG received habitual education, consisting in a pre-operative nursing consultation, where verbal information was provided on the characteristics of the intervention; a clinical interview; and education about self-care. The EG additionally received a tryptic with detailed information about the preparation of the surgery and recommendations for managing post-operative pain. Characteristics of the sample and follow up: 497 patients over 16 years of age, without difficulties for communication or cognitive impairment. The CG patients were intervened from July 2006 to June 2007. The EG patients were intervened between July 2007 and June 2008. Data were collected upon admission to the Ambulatory Surgery Unit, where the degree of understanding of the information received was measured and ended with a phone call 24 hours post-operative to assess the degree of pain. Result measurements: VAS. Results: The CG patients who received the usual education plus a tryptic with detailed information about the preparation of the surgery and recommendations for managing postoperative pain manifested pain in 73.2% of the cases, against 78.1% in the experimental group (p = 0.2), with this being an insignificant difference. However, the difference of patients with a VAS > 3 between the control and experimental groups was statistically significant. Conclusions: An educational intervention conducted by nurses, based on the delivery of graphic material to manage postoperative pain in ambulatory hernia surgery, achieves lower pain in the first 24 hours postoperative, having less problems in mobilization, and greater adherence to treatment with analgesics. Gender or age of patients can influence on the perceptions of pain. In the clinical practice, these types of interventions could be useful to improve therapeutic adherence. Quality score: JBI scale for Quasi-experimental Studies 8/9.
23	Authors, year: Reynolds; 2009. Country: The United States. Type of study: Quasi-experimental study. Objective: To analyze the effectiveness of an educational intervention on the degree, knowledge, attitude, management, and satisfaction of pain in the daily lives of patients after one week of discharge after a surgery. Intervention: The CG had 59 patients who received habitual information. The EG had 87 patients who received the habitual information implemented with a brochure that added information that covered topics about the beliefs about pain, how to recognize the barriers related with its treatment, how to seek information, and the importance of staying in contact and conducting the follow up with the health service. Characteristics of the sample and follow up: 146 patients over 18 years of age from two rural hospitals, who could communicate in English, without cognitive impairment and capable of filling out the study questionnaire. The variables to evaluate were measured before the randomization of the groups and patients were followed up one week later. Result measurements: Numerical Graduation Scale of pain. Brief Pain Inventory. Results: The results of this study show that, during the pre-test, just before the educational intervention, both the CG and the EG had the same thoughts about pain and its management and both indicated they would experience from 7 to 10 points of pain after the surgery. After the surgery, there were significant differences in the sensation of pain between both groups, However, there were pain scores above 6 points in 7% of the patients (12% in the CG, 5% in the EG). In all, 81% of the patients manifested that the nurse clarified the importance of the treatment and were satisfied with pain management by the nursing staff. Conclusions: An educational intervention based on delivering a brochure with information about topics on pain, the way of solving barriers for its treatment or the importance of follow up with health services for its management can be effective to reduce postoperative pain perceived by patients. This study shows the usefulness of providing adequate information and including the patients within the treatment as active subjects. Hence, future studies should delve into these aspects. Quality score: JBI scale for Quasi-experimental Studies 7/9.
24	Authors; year: Van Dijk et al.; 2015. Country: Holland. Type of study: Quasi-experimental study. Objective: To analyze the effect of a pre-operative educational intervention on the demand for opioids by patients, and know the repercussion of the intervention on post-operative pain, fears, and knowledge about opioids. Intervention: The CG had 183 patients who watched a 3-minute film that explained how the hospital’s information and entertainment system operated. The EG had 194 patients who were shown a 6-minute film where the actors explained through interpretation the Numerical Graduation Scale, the importance of managing post-operative pain, and the to move and cough to prevent complications. Characteristics of the sample and follow up: 357 adult patients capable of communicating and understanding German. The variables to study were gathered after watching the film, measuring beliefs about pain, as well as the demographic variables. The level of pain was again measured 24 hours after the surgery. Result measurements: Numerical Graduation Scale of pain and Verbal Rating Scale (VRS) to assess pain. Anxiety was measured through the Questionnaire on Fear of Surgery from Holland. Results: No significant differences were noted on the perception of pain in both groups after the intervention, although the patients in the experimental group reflected greater knowledge and lesser difficulty when facing post-operative pain. Lower levels of pain were shown in the EG due probably better understanding of it. Additionally, they did not require extra analgesia. Better knowledge of opioids helped to improve the process of patient-professional communication. Conclusions: An educational intervention based on viewing a film about the characteristics of pain and the importance of its treatment is capable of reducing pain in patients subjected to surgery, additionally, helping to increase their knowledge about analgesia and reducing barriers in the treatment. In the clinical practice, this intervention could aid in patients expressing more precisely their pain, being useful to professionals to make better decisions. Quality score: JBI scale for Quasi-experimental Studies 6/9.
25	Authors; year: Chen et al.; 2014. Country: China. Type of study: Quasi-experimental study. Objective: To evaluate the effectiveness of an educational intervention on post-surgical pain, rehabilitation, and functional recovery of patients intervened for knee replacement. Intervention: The CG had 50 patients. The EG had 42 patients who received an informative intervention based on the delivery of a pamphlet and a CD with detailed information and examples of pre- and postoperative care, rehabilitation exercises, and mobilization methods. Characteristics of the sample and follow up: 92 patients 18 years of age or older, intervened for the first time for knee replacement that could move about and get out of bed before the operation, and free from post-operative complications. The variables were measured the day before the intervention and daily the first five days after surgery. Result measurements: Numerical Graduation Scale of pain for pain. Besides, functionality was evaluated with the Chinese adaptation of a functional state subscale of the Multidimensional Functional Evaluation Questionnaire. Results: In general, pain perceived by the experimental group, which received an educational intervention based on the delivery of an informative pamphlet plus an explicative CD, was significantly lesser. The differences in the measurements of perceived pain were statistically significant in the first three days after surgery. In addition, good results were found in the performance of the rehabilitation and acquisition of strength. However, no differences were found between groups in functional recovery. Conclusions: A pre-operative educational intervention supported by the use of an educational brochure and a CD can reduce the level of postoperative pain experienced by patients with total knee replacement, as well as increase the regularity of rehabilitation exercises and muscular strength of the affected leg. In the clinical practice, this type of intervention could increase the regularity of performing rehabilitation exercises, making the rehabilitation more effective. Quality score: JBI scale for Quasi-experimental Studies 8/9.

Regarding the principal results reported in these studies after these interventions, while certain studies found a significant reduction of post-surgical pain after the educational intervention,(14,15,25) other did not report statistically significant reduction of pain after the intervention.(16,17,20,22-24) In these last studies, although no significant differences were reached, the levels of pain in the experimental group were lower. Additionally, all those studies that analyzed anxiety found statistically significant differences in the levels of anxiety between the groups after the educational intervention.(15,17,20,21) 

Studies inquiring on the interference of pain in the activities of daily life found that, after the educational intervention, pain interfered less in the experimental group, which is why these patients had less problems to mobilize, walk, or perform actions, like coughing.(16,18,21-23,25) Other results reported in the studies were that the educational intervention increased the participants’ knowledge about analgesics, as well as their consumption, improving adherence treatment after the intervention.(22) Besides, greater therapeutic adherence permitted better control of pain and with it less problems in mobility.(22) In another study, the educational intervention helped in the functional improvement and rehabilitation of the member affected,(25) while a study reflected that the educational intervention had no effect on the patient’s vital constants (cardiac frequency, blood pressure, and respiratory frequency).(15) 

In relation to the methodological quality of the studies analyzed, it was found that the six RCT, evaluated with the JADAD scale,(12) obtained scores of 2 points,(14,16) 3 points(15,17,19), and 4 points from a maximum of 5 points.(18) The study evaluated with the Joanna Briggs Institute Checklist for Systematic Reviews(11) reached a score of 8 points from a maximum of 11 points;(20) and the five quasi-experimental studies evaluated with the Joanna Briggs Institute Checklist for Quasi-experimental Studies,(13) obtained scores of 6 points,(24) 7 points(21,23) and 8 points (22,25) over a maximum score of 9 points.

## Discussion

The results of the studies included in this systematic review do not permit generalizing that the educational interventions aimed at patients for the knowledge of pain and its management to be effective in controlling or reducing post-surgical pain, due to the controversy found in the results. However, the results found show greater pain relief in patients receiving these interventions, as well as lower incidence of pain in performing daily activities after the intervention, like mobilization, walking, or resting. Additionally, educational interventions improve the expectations of patients on post-surgical pain, modify negative preconceptions of opioid analgesics, and improve the use of analgesics during the post-operative period. Regarding prior studies that have only analyzed the association between the educational interventions and relief of post-surgical pain, this review contributes with the analysis of the benefits of the educational interventions to perform daily activities after surgery (16,18,21-23,25) or the relationship between the educational interventions and the vital constants (cardiac frequency, respiratory frequency, and blood pressure) of post-operative patients.(15)

Following the line of prior studies, most of the articles analyzed coincide in that the information provided during the educational intervention is an important tool to raise awareness and sensitize patients on the concept of pain and the need for its treatment, producing a behavioral change in patients toward this pain.(15-18,20,25) As shown in other studies, the results suggest a change, after the educational intervention, in the expectations of patients on post-surgical pain and modification of prior negative conceptions about opioid drugs used in pain management.(22-24) In this sense, the results from this review show that patients’ increased knowledge improves its inclusion in the treatment, providing patients an active role, reducing their fear, anxiety, barriers, prejudice, and beliefs when using analgesics.(15,16,22-24) 

However, although prior studies show the benefits of the educational interventions, certain studies in this review do not confirm this fact, not finding that the educational intervention presents any repercussion on relieving post-surgical pain or on the interaction of pain in daily activities.(16,17,20,22-24) Although most of the studies suggest that the educational intervention can be useful in managing post-surgical pain, said association cannot be confirmed because not all the studies found in this review are conclusive, and some articles lack sound scientific evidence to ensure the results. 

The results from this review show that educational interventions carried out by nursing can improve relief of post-surgical pain.(22) In addition, nursing educational interventions reduce mobility problems, improve adherence to treatment after surgery(22), and increase patient satisfaction.(23) Nursing interventions aimed at increasing patients’ knowledge about analgesia before the intervention help the person identify and control pain, thus, reducing possible barriers during treatment.(23,25) During the clinical practice, educational interventions help patients to assume an active role, allowing them to express their pain more precisely and learn that therapeutic alternatives exist.(23,25) Hence, educational interventions are a tool that help nursing professionals to improve the process of making shared decisions, adherence to treatment, and management of post-surgical pain.(22)

The principal strength of this review is that it has followed the recommendations of the PRISMA Declaration and has evaluated the quality of the studies included with different instruments, according to the type of study. The study had mainly two limitations: the first is that of having found few studies with not very high level of rigor and very diverse applied methodology, and the second, is that considering only articles published in Spanish or English in the databases analyzed constitutes a limitation, when excluding possible relevant articles published in other languages. 

The conclusion of this review is that educational interventions could influence on the relief of post-surgical pain, as well as aid in the development of daily activities, like moving about or breathing. Furthermore, educational interventions improve management of post-surgical anxiety and reduce barriers during the treatment follow up. In spite of the aforementioned, more rigorous studies are necessary, with larger sample sizes and higher methodological quality, which help to learn with certainty the effect of educational interventions in managing post-surgical patients with pain and extract relevant information that permits developing new pre-operative protocols that help to reduce pain and its post-operative complications. 
